# Improving communication for immunisation in Africa: contribution of the Vaccines for Africa website

**Published:** 2009-04-14

**Authors:** Charles Shey Wiysonge, Zainab Waggie, Linda Rhoda, Gregory Hussey

**Affiliations:** 1 School of Child and Adolescent Health and Institute of Infectious Disease and Molecular Medicine, Faculty of Health Sciences, University of Cape Town, South Africa

**Keywords:** Immunization, Africa, Communication strategies

## Abstract

**About the authors:**

C. Wiysonge is a medical epidemiologist and Vaccinology Programme Manager at the Institute of Infectious Disease and Molecular Medicine, University of Cape Town, South Africa. He previously worked for the Expanded Programme on Immunisation in Cameroon and has been a consultant on vaccines and immunisation for WHO and the GAVI Alliance. Z. Waggie is a Paediatrician and Senior Clinical Research Officer at the Institute of Infectious Disease and Molecular Medicine, University of Cape Town, South Africa. L. Rhoda is a communications specialist and Communications Manager at the South African Tuberculosis Vaccine Initiative, University of Cape Town, South Africa. G. Hussey is a Paediatric Infectious Diseases Clinical Specialist and Professor of Child and Adolescent Health, Director of the Institute of Infectious Disease and Molecular Medicine, and Director of the South African Tuberculosis Vaccine Initiative, University of Cape Town, South Africa. He has been a WHO part-time consultant on vaccines and immunisation for the past 10 years.

## Editorial

Immunisation is the most cost effective public health discovery and one of the greatest medical achievements of the 20th century, saving more lives than any other health care intervention. It is estimated that immunisation against vaccine preventable diseases saves over two million lives each year [1]. There are currently more than 25 vaccine-preventable diseases and the number of diseases that can be prevented by vaccines is growing. Advances in biomedical research, science, and technology are making this possible. Vaccines thus provide an effective, economical, and practical way of preventing disease and disability, and promoting health and well being. Immunisation protects not only the individual but also the wider community by reducing the probability of the transmission of infection in the community [2].

The introduction of immunisation programmes worldwide, following the initiation of the Expanded Programme on Immunisation (EPI) in 1974 by the World Health Organization (WHO), has resulted in significant accomplishments. In 1977, smallpox was eradicated after a 10-year campaign [1]. When the Smallpox Eradication Programme began, the disease threatened 60% of the world’s population and killed every fourth person infected. Now, poliomyelitis is the next disease in line to be eradicated. Since the Global Polio Eradication Initiative was launched in 1988, the incidence of polio has decreased by more than 99% and about five million people have escaped paralysis by the wild poliovirus [1, 3]. Today, polio is endemic in only four countries in the world. The active surveillance system for acute flaccid paralysis, within the context of the Global Polio Eradication Initiative (PEI), has contributed to the improvement of health information management systems within countries including the integration of surveillance for other vaccine-preventable diseases, including measles. WHO estimates that measles deaths worldwide fell by a remarkable 74% between 2000 and 2007 from 750,000 to 197 000. It is estimated that during this period, 11 million measles deaths were averted globally as a result of measles control activities [4]. Furthermore, childhood vaccination coverage in general has increased dramatically in the past two to three decades. In 2007, global coverage for three doses of the diphtheria-tetanus-pertussis (DPT) combination vaccine was 81%; up from 20% in 1980 [1].

However, despite these tremendous advances, more than 24 million children still do not have access to basic immunisation services, including 7.3 million in sub-Saharan Africa [1]. Those who miss out on routine vaccination programmes tend to live in remote locations, urban slums, and border areas. Such hard-to-reach populations also include displaced populations, those lacking access to vaccination because of various social barriers, those lacking awareness or motivation to be vaccinated, and those who refuse to be vaccinated. The consequence of not having access to vaccines, or not being immunised, is the untimely death of between two and three million people each year from vaccine-preventable diseases [1]; a large proportion of these deaths occur in Africa.

In general, Africa lags behind other continents in the uptake of life-saving vaccines even though vaccine-preventable diseases are causing millions of untold and avoidable deaths across the continent. Most children in Africa have access to only six to eight vaccines. In contrast, a child born in a high-income country such as the United States has access to more than 15 vaccines [5]. This vaccine gap is widening by the day. There is an urgent need to reduce morbidity and mortality from vaccine-preventable diseases in Africa by increasing awareness about the benefits of vaccines and promoting the uptake of both existing and new vaccines, increasing coverage of existing EPI vaccines, and advocating for the inclusion of new vaccines in EPI programmes. It is encouraging to note that the South African Health Department has introduced rotavirus vaccine and the conjugated pneumococcal vaccine into its national EPI programme, a move that will significantly reduce childhood morbidity and mortality in the country.

Effective communication should be an essential component of the EPI in Africa, because it could mobilise resources for national immunisation programmes, encourage wide participation and ownership of immunisation services among all stakeholders in each African country, and lead to positive changes in knowledge and attitudes towards immunisation in Africa.

These are the reasons why the Vaccines for Africa (VACFA) website (www.vacfa.com) was launched on 31 March 2009. The website is intended to be an interactive forum for the exchange of accurate, up-to-date, and evidence-based information on vaccines and immunisation practices relevant to Africa. As such, it would be a “One-Stop-Shop” for targeted information which responds to the information needs of health professionals, policymakers, programme managers, and the public. VACFA complements the work of international immunisation advocacy organisations such as WHO, the United Nations Children’s Fund, GAVI Alliance and other agencies in bringing the right information to the right people at the right time in order to ensure that right decisions are made in a timely manner.

## Competing interests

None declared
